# Supplementary material for: Subjective valuation as a domain-general process in creative thinking
Source: Commun Psychol. 2025 Jul 17;3:108. doi: 10.1038/s44271-025-00285-8 (PMC12271468; doi:10.1038/s44271-025-00285-8)
Supplement: Supplementary file 2 — SupplementaryInformation [file 44271_2025_285_MOESM2_ESM.pdf]

Supplementary Information

Supplementary Notes

Supplementary Note 1 | Relationship between orthogonalized likeability ratings and RT and PS

As likeability is correlated with adequacy and originality ratings (see Results), we orthogonalized likeability ratings for adequacy and originality. We found that relationships were still significant in all domains for response time (RT), and similar to the main finding for production speed (PS) (Supplementary Table 1).

Supplementary Note 2 | Relationship between response time and proxy for confidence

We checked whether response time could capture a form of confidence [1] instead of satisfaction with the response. To do so, we built a mixed model integrating the condition (*First* versus *Creative*) with squared and first power likeability ratings to explain response time. We found that the quadratic term did not significantly explain response time compared to the linear term (Supplementary Table 2). In other words, by squaring likeability, we add to the model a variable testing for confidence encoding. Thus, this added variable has no significant correlational relationship with response time.

Supplementary Note 3 | Uses domain – Replication of results with an alternative scoring method.

We conducted a control analysis by using an alternative scoring method (Open Creativity Scoring with Artificial Intelligence, OSCAI[2]), which provide two scores per response : originality and elaboration ($OSCAI_{Originality}$ and $OSCAI_{Elaboration}$). We found a significant correlation between SemDis and OCSAI scores ($r_{OCSAIOriginality,SemDis}(70)=0.20$, p=$\mathbf{6.10^{-72}}$, 95% CI=[0.18,0.22]; $r_{OCSAIElaboration, SemDis}(70)=0.47$, p$\mathbf{<1.10^{-6}}$, 95% CI=[0.46,0.49]), with qualitatively

and quantitatively similar results from the analyses conducted with SemDis scores (Supplementary Figure 4). The results indicate that participants' responses were more original and more elaborate in the *Creative* condition compared to the *First* condition (Uses ($OCSAI_{Originality}$): $Prediction_{First}$=1.151±0.009, $Prediction_{Creative}$=2.441±0.044, t(72)=28.18, p=**1.10^{-40}**, d=4.72, 95% CI=[1.20,1.39]; Uses ($OCSAI_{Elaboration}$): $Prediction_{First}$ =2.920±0.162, $Prediction_{Creative}$ =4.021±0.188, t(72)=6.48, p=**1.10^{-8}**, d=0.83, 95% CI=[0.02,0.03]).

Then, regarding the canonical correlation analysis, the first canonical variable raised a significant correlation (r(70)=0.48, Wilks statistic=0.64, F(18,178.7)=1.67, p=**0.048**). All variables of the Questionnaire Set contributed significantly to its associated canonical variable (Questionnaire set: $r_{C\text{-}Ach}$(70)=0.95, p=**1.10^{-35}**, 95% CI=[0.91,0.97]; $r_{C\text{-}Act}$(70)=0.63, p=**4.10^{-9}** , 95% CI=[0.46,0.75]; $r_{C\text{-}self\text{-}report}$(70)=0.73, p=**$4.10^{-13}$**, 95% CI=[0.60,0.82], Pearson correlation coefficient between variables and canonical variable of the set). In the Model & Behavior set, the model's parameters (r_{α}(70)=0.24, p=**0.038**, 95% CI=[0.01,0.45]; r_{δ}(70)=-0.32, p=**0.007**, 95% CI=[-0.51,-0.09]) contributed significantly to their associated canonical variable. Regarding automatic scoring of creativity, both the AuDra score ($r_{Drawings\text{ - }AuDra}$(70)=0.81, p=**$7.10^{-18}$**, 95% CI=[0.71,0.88]) and the Word2Vec score (negative cosine similarity) ($r_{Words\text{ - }Word2Vec}$(70)=0.64, p=**$1.10^{-9}$**, 95% CI=[0.48,0.76]) contributed significantly and positively to the associated canonical variable. OCSAI scores did not show any significant contribution ($r_{Uses\text{ – }OCSAI\text{ (Originality)}}$(70)=0.04, p=0.763, 95% CI=[-0.20,0.27]; $r_{Uses\text{ – }OCSAI\text{ (Elaboration)}}$(70)=0.18, p=0.137, 95% CI=[-0.06,0.39]).

Supplementary Note 4 | Drawing domain – Estimation of AuDra reliability with human expert ratings.

To confirm the reliability of AuDra scores, we used the consensual assessment technique recommended for evaluating creativity tasks[3]. For each of the 30 abstract shapes used as cues, we selected four participants' drawings based on the quantile distribution of the AuDra score for those abstract shapes. This method ensures a balanced diversity of AuDra scores for each cue. Next, seven judges from the lab, all experienced in creativity assessments but

unfamiliar with the data, were recruited. The judges first viewed the 30 abstract shapes, each followed by the four selected drawings. After this initial viewing, the 120 selected drawings were presented in a randomized order, and judges rated them on a scale from 0 (not creative at all) to 4 (extremely creative). Intraclass correlation estimates indicated good reliability among judges' ratings (ICC=0.858, 95% CI= [0.816 0.893]), with 95% confidence intervals, based on a mean-rating (k=7), absolute agreement, and 2-way random-effects model. A Spearman correlation analysis between the mean of the judges' ratings and the AuDra scores revealed a significant positive relationship (r(118)=0.47, p<$\mathbf{1.10^{-3}}$, 95% CI=[0.31,0.60]).). Note that in Patterson et al (2024)[4], the correlation between AuDra scores and human raters for drawings that do not include the original abstract shapes on which the model was trained yielded similar results (r=0.49, 95% CI=[0.43, 0.54]).

Supplementary Methods

Supplementary Method 1 | Task stimuli

Free Generation of Associates Task - Words task. Cue words were nouns with a lexical frequency of over 15 occurrences per million and a number of syllables lower than four. All this information was extracted from http://www.lexique.org/. Half of the cues were defined as steep (with a strongly dominant associate) and the other half flat (more balanced strength with its associates).

- Steep cues: (**FR**) avis, doigt, appel, sens, page, lutte, façon, chien, vache, rayon, rose, jeunesse, soeur, question, flamme; (**EN**) notice, finger, call, meaning, page, fight, way, dog, cow, ray, pink, youth, sister, question, flame.
- Flat cues: (**FR**) arbre, fil, horizon, mur, preuve, vin, pierre, jardin, faim, cause, habitude, jambe, morceau, machine, lune; (**EN**) tree, thread, horizon, wall, proof, wine, stone, garden, hunger, cause, habit, leg, piece, machine, moon.

Free Generation of Alternate Uses Task - Uses task. Cue words were the object's name, extracted from the previous protocol with the Alternate Uses of Object Task [5–7].

- (**FR**) pneu, couteau, bouteille, ceinture, brique, balai, seau, bougie, horloge, peigne, lampe, stylo, oreiller, sac, chaussette, ballon, chapeau, briquet, rasoir, écharpe, corde, pelle, fourchette, table, pantalon, chaussure, livre, trombone, serviette, journal; (**EN**) tire, knife, bottle, belt, brick, broom, bucket, candle, clock, comb, lamp, pen, pillow, bag, sock, balloon, hat, lighter, razor, scarf, rope, shovel, fork, table, pants, shoe, book, paper clip, towel, newspaper

Free Generation of Drawings Task - Drawings task. Cue shapes were abstract forms extracted from Nishimoto et al. (2010) [8] based on the H metrics (i.e., level of disagreement, an equivalent of flatness). Stimuli that were not centered, evoking cultural facts, or too similar between them, were not selected.

- L07, L15, L16, L18, L27, L35, L36, L43, L47, L48, L49, L59, L63, L65, L67, L68, L74, L84, L86, L93, L97, R09, R12, R40, R45, R51, R54, R90, R94, R98

Supplementary Method 2 | Additional associations presented in the rating tasks

In rating tasks, potential responses rated by participants comprised their responses from the *First* and *Creative* conditions, as well as other potential responses to create additional cue-response associations. We detail here how these additional associations were built.

Uses task:

Before their use in the protocol, the cues of the Uses task were paired with common uses and uncommon uses that we invented. Then, each association was independently scored before data collection by 5 experts. The judges were instructed to give each association a creativity score between 0 (not creative at all) and 5 (highly creative). Common associations (i.e., equivalent to the *First* condition) have a mean score of 1 across judges, and uncommon associations (i.e., equivalent to the *Creative* condition) have a mean score of creativity of 4.2.

Words Task:

For the Words task, additional associations presented during the rating tasks were pseudo-randomly built from an independent dataset (of the Words task) and constrained by the following rules for each cue:

- A frequent response from the *First* condition (A) was randomly selected among the most frequent responses of our independent dataset.

- An infrequent response from the *First* condition (B) was randomly selected among the rarest responses of our independent dataset.
- A frequent response from the *Creative* condition (C) was randomly selected among the most frequent responses of our independent dataset.
- An infrequent response from the *Creative* condition (D) was randomly selected among the rarest responses of our independent dataset.
- An unrelated word (E) that had no evident semantic link to any of the cue words. We created 30 cue-unrelated word associations that were the same for all participants.

As each cue is then associated with seven potential responses (the *First* and *Creative* responses from the subject and the five additional associations), it gives a total of 210 associations. To reduce the number of ratings, we selected the 22 most representative associations A, B, C and D (the most frequent for A and C and the most infrequent for B and D), yielding to a maximum of 118 additional associations (on top of the participants responses), so a total of 178 ratings maximum per participant. Some participants rated fewer associations because their responses with misspelling where automatically removed from the list of responses to rate. In the end, the average number of word associations rated was 131, ranging from 59 to 171.

Drawings Task:

For the Drawing task, additional drawings were selected based on a pilot of the task on four participants, comprising 20 abstract shapes. For each abstract shape, we selected one *First* response and one *Creative* response from the pilot participants, based on the quality of the drawings. In addition, 20 abstract drawings were designed by the experimenter. Then, a total of 60 additional drawings were presented to the participants in the rating tasks (20 *First*, 20 *Creative*, 20 abstracts).

Supplementary Method 3 | Recovery analysis of the CES model

To evaluate the accuracy and reliability of the model's parameter estimation, we performed a recovery analysis of the constant elasticity of substitution (CES) value function parameters on the data of 20 participants selected randomly, to minimize computational cost. The process can be divided into the following steps:

1) *Define the range of value for α and δ parameters (predefined parameters).*

For each parameter, we generated 20 predefined values from -10 to 10, constrained between 0 to 1 after softmax transform for α, and from -5 to 5 for δ.

2) *Define all possible combinations of a priori α and δ.*

We built the 400 possible combinations of predefined parameters α and δ.

3) *For each combination, simulate likeability ratings.*

For each combination and participant, adequacy and originality ratings were kept, and the likeability ratings were simulated based on the predefined value of parameters and participant ratings.

4) *Performed model inversions for parameter estimation.*

Then, we fitted the CES model on simulated likeability ratings. This fitting was done for each participant and each possible combination of predefined α and δ parameters for likeability simulation. The same model fitting was made with 30% of noise added to simulated likeability to improve the ecological validity of this analysis.

5) *Recovery of the predefined parameters.*

We tested the correlation between the 400 values of predefined and estimated parameters and reported the results in Supplementary Figure 3.

Supplementary Figures

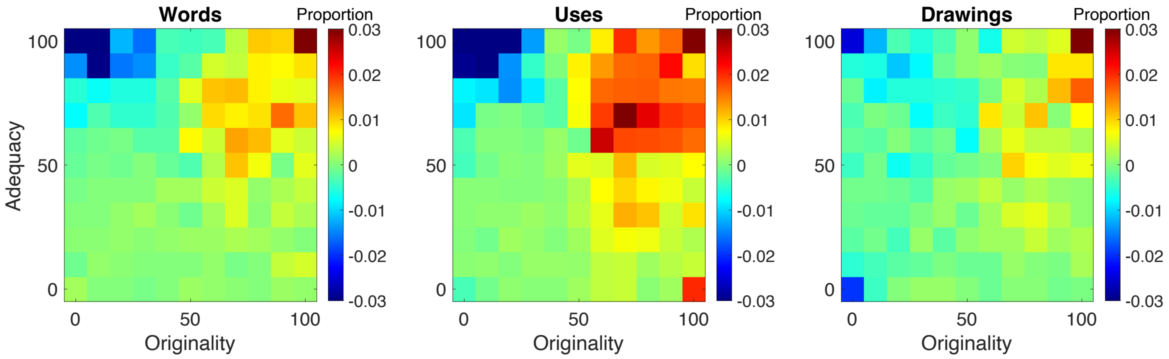

Supplementary Figure 1 | Proportion of responses for generation tasks according to their adequacy and originality. Heatmaps of *Creative*-*First* proportions of responses per bin of adequacy and originality ratings are displayed for each task. Color code indicates a difference of proportion between the *Creative* and *First* conditions. Within each heatmap, the maximum proportion is located at the top-right corner (i.e., highly original, highly adequate associations, in *Creative* condition), and the minimum proportion is located at the top-left corner (i.e., slightly original, highly adequate associations, in *First* condition). All participants were included in those analysis (n=73).

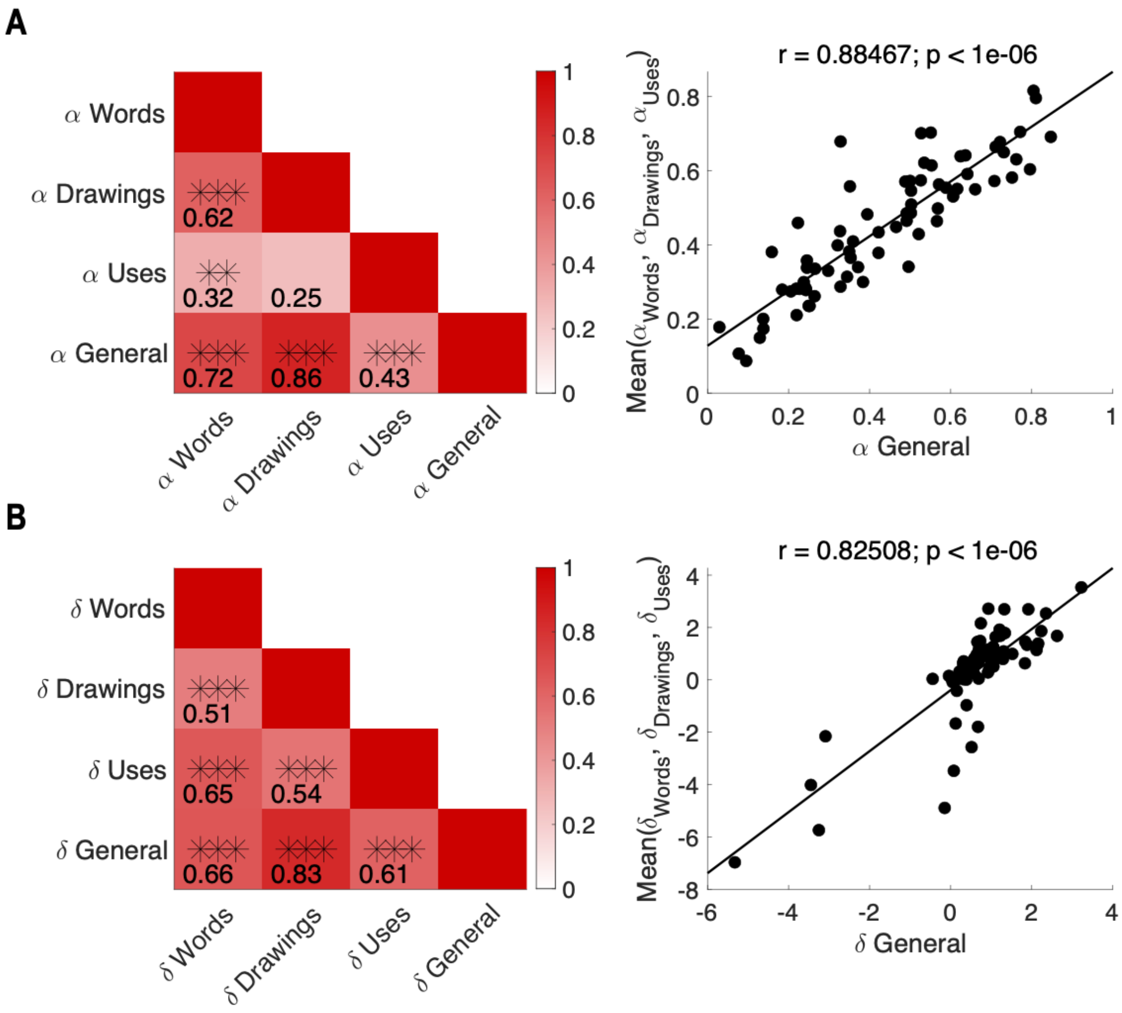

Supplementary Figure 2 | Domain-general and domain-specific model parameter correlations. (A) One-tailed Pearson's correlation matrix for the (left) and scatter plot between the general value, estimated on pooled data, and the mean of the α parameters estimated from the three domains separately. **(B)** Same for the δ parameter. Only significant p-values that survive after Bonferroni correction are displayed. All participants were included in those analysis (n=73).

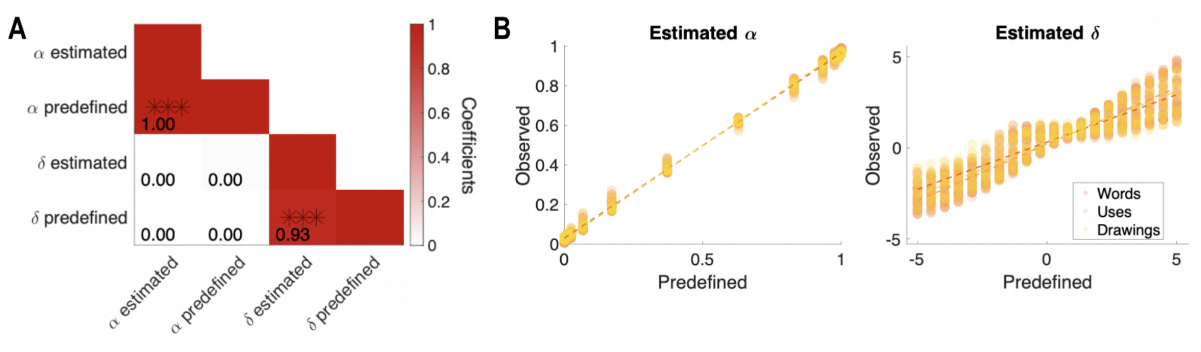

Supplementary Figure 3 | Recovery Analysis. (**A**) Correlation matrix between estimated α and δ parameters of the CES models and their associated predefined values. For simulations, different values of α (ranging from -10 to 10, constrained with softmax function between 0 and 1) and δ (ranging from -5 to 5) were used to design 400 combinations of these parameters. Then, model inversions were performed for parameter estimation on simulated likeability ratings and observed adequacy and originality ratings. 30% of noise was added to simulate the likeability rating. (**B**) Respective correlation between predefined parameter's value (after softmax transform for α) and observed value from parameter estimation.

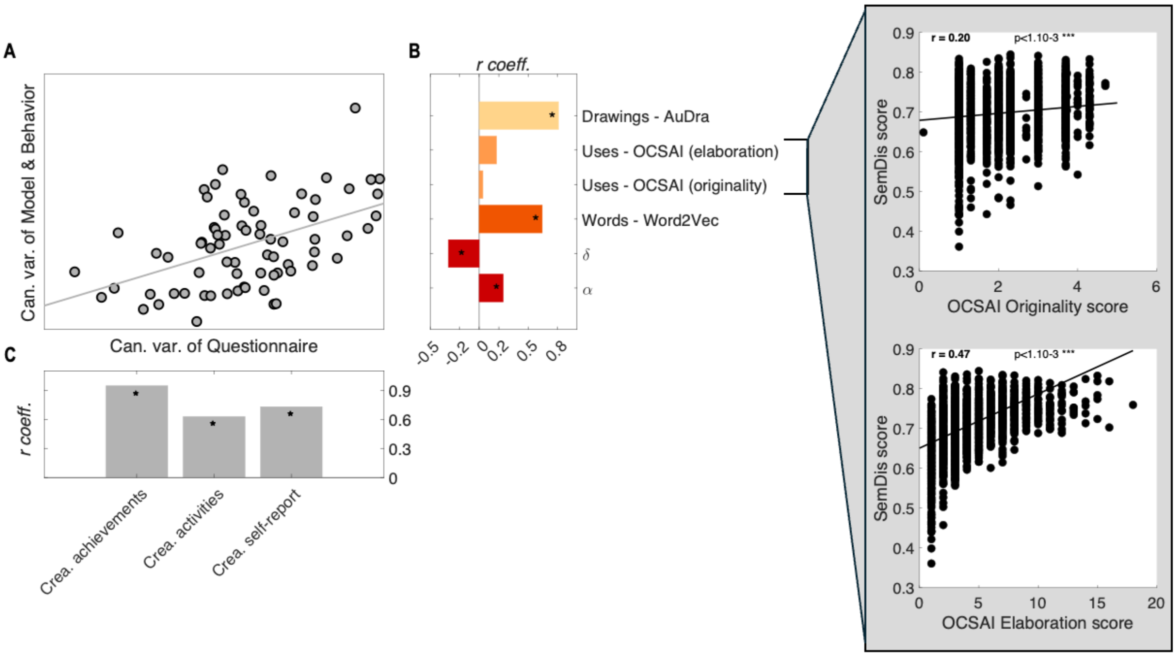

Supplementary Figure 4 | Canonical correlation between Model & Behavior set and Questionnaire set – OSCAI scoring for the Uses domain. (**A**) Correlation between the first canonical variable of the Questionnaire set and the first canonical variables of the Model & Behavior set. (**B**) Bar plots represent the correlation coefficients between each variable of the Model & Behavior set with the first canonical variable from the Model & Behavior set. For Words, Uses, and Drawings, variables are individual means of respective scores. (**C**) Same as **(B)** for each variable of the Questionnaire set with the first canonical variable from the Questionnaire set. Stars indicate the significance of the correlation ($p<0.05$). (**Zoom panel**) Spearman correlation between mean OCSAI originality and elaboration scores with SemDis score across participants. All participants were included in those analysis (n=73).

Supplementary Tables

Response time	zscored					zscored & orthogonalized			
	**Mean**	**SEM**	**t-stats**	**p-value**	**df**	**Mean**	**SEM**	**t-stats**	**p-value**
**Words**	-0.113	0.025	-4.454	3.10^{-5}	72	-0.06	0.023	-2.647	0.010
**Uses**	-0.169	0.024	-7.103	8.10^{-10}	71	-0.141	0.024	-6.003	7.10^{-8}
**Drawings**	-0.248	0.027	-9.029	2.10^{-13}	72	-0.07	0.025	-2.923	5.10^{-3}

Production speed	zscored					zscored & orthogonalized			
	**Mean**	**SEM**	**t-stats**	**p-value**	**df**	**Mean**	**SEM**	**t-stats**	**p-value**
**Words**	0.132	0.028	4.683	1.10^{-5}	72	0.074	0.028	2.694	0.009
**Uses**	0.243	0.028	8.506	2.10^{-12}	71	0.169	0.028	6.027	7.10^{-8}
**Drawings**	-0.016	0.025	-0.613	0.542	72	0.020	0.024	0.855	0.395

Supplementary Table 1 | Detailed results of the regression coefficients for response time and production speed against likeability and orthogonalized likeability in the *Creative* conditions of the three domains. Results of two-tailed one-sample t-test against 0 for the likeability and likeability orthogonalized for adequacy and originality ratings.

						95% CI	
**Name**	**Estimate**	**SE**	**tStat**	**df**	**p-value**	**Lower**	**Upper**
Intercept	3.263	2.944	1.108	11838	0.268	-2.508	9.035
Condition	6.763	0.539	12.559	11838	6.10^{-36}	5.708	7.819
Likeability rating	-0.003	2.733	-0.001	11838	0.999	-5.362	5.356
Likeability rating^2	5.10^{-4}	2.752	2.10^{-4}	11838	0.999	-5.395	5.396
Condition*Lik	-0.037	0.009	-4.051	11838	5.10^{-5}	-0.055	-0.019
Condition *Lik^2	5.10^{-4}	3.10^{-4}	1.532	11838	0.125	-1.10^{-4}	1.10^{-3}
Lik*Lik^2	-6.10^{-6}	3.10^{-6}	-1.577	11838	0.115	-1.10^{-5}	1.10^{-6}
Condition *Lik*Lik^2	-2.10^{-6}	5.10^{-6}	-0.519	11838	0.604	-1.10^{-5}	7.10^{-6}

Supplementary Table 2 | Summary of model's statistics. Results of mixed-model analysis based on the following formula (L stands for Likeability):

$$RT \sim L * L^2 * cond + (1|task) + (-1 + L|task) + (-1 + L^2|task) + (1|subject_{ID}) + (-1 + L|subject_{ID}) + (-1 + L^2|subject_{ID})$$

Model ID	Model name	Model description	Number of free parameters	Ef	Xp
**1**	All ~	all parameters are different between domains	6	0.001	0.000
**2**	δ ~	α are equal between domains<br>δ are different between domains	4	0.001	0.000
**3**	$\alpha_{Words}=\alpha_{Drawings}$	α are equal between Words and Drawings tasks only<br>δ are different between domains	5	0.001	0.000
**4**	$\alpha_{Words}=\alpha_{Uses}$	α are equal between Words and Uses tasks only<br>δ are different between domains	5	0.001	0.000
**5**	$\alpha_{Drawings}=\alpha_{Uses}$	α are equal between Drawings and Uses tasks only<br>δ are different between domains	5	0.001	0.000
**6**	α ~	α are different between domains<br>δ identical	4	0.176	0.000
**7**	$\delta_{Words}=\delta_{Drawings}$	α are different between domains<br>δ are equal between Words and Drawings tasks only	5	0.001	0.000
**8**	$\delta_{Words}=\delta_{Uses}$	α are different between domains<br>δ are equal between Words and Uses tasks only	5	0.001	0.000
**9**	$\delta_{Drawings}=\delta_{Uses}$	α are different between domains<br>δ are equal between Drawings and Uses tasks only	5	0.001	0.000
**10**	Words=Drawings	α and δ are equal between Words and Drawings tasks only	4	0.001	0.000
**11**	Words=Uses	α and δ are equal between Words and Uses tasks only	4	0.001	0.000
**12**	Drawings=Uses	α and δ are equal between Drawings and Uses tasks only	4	0.070	0.000
**13**	All =	α are equal between domains<br>δ are equal between domains	2	**0.742**	**1.000**

Supplementary Table 3 | Model space of the CES value function explaining likeability ratings in the three domains. Symbols ~ and = respectively stand for different and equal between domains. Model n°1 is the full model (i.e., all parameters are different between tasks), and model n°13 is the reduced model (i.e., all parameters are identical between tasks).

Supplementary references

1. Lebreton, M., Jorge, S., Michel, V., Thirion, B. & Pessiglione, M. An Automatic Valuation System in the Human Brain: Evidence from Functional Neuroimaging. *Neuron* **64**, 431–439 (2009).
2. Organisciak, P., Acar, S., Dumas, D. & Berthiaume, K. Beyond semantic distance: Automated scoring of divergent thinking greatly improves with large language models. *Think. Ski. Creat.* **49**, 101356 (2023).
3. Ceh, S. M., Edelmann, C., Hofer, G. & Benedek, M. Assessing Raters: What Factors Predict Discernment in Novice Creativity Raters? *J. Creat. Behav.* **56**, 41–54 (2022).
4. Patterson, J. D., Barbot, B., Lloyd-Cox, J. & Beaty, R. E. AuDrA: An automated drawing assessment platform for evaluating creativity. *Behav. Res. Methods* **56**, 3619–3636 (2024).
5. Beaty, R. E., Johnson, D. R., Zeitlen, D. C. & Forthmann, B. Semantic Distance and the Alternate Uses Task: Recommendations for Reliable Automated Assessment of Originality. *Creat. Res. J.* **34**, 245–260 (2022).
6. Maio, S., Dumas, D., Organisciak, P. & Runco, M. Is the Reliability of Objective Originality Scores Confounded by Elaboration? *Creat. Res. J.* **32**, 201–205 (2020).
7. Stevenson, C., Baas, M. & van der Maas, H. A Minimal Theory of Creative Ability. *J. Intell.* **9**, 9 (2021).
8. Nishimoto, T., Ueda, T., Miyawaki, K., Une, Y. & Takahashi, M. A normative set of 98 pairs of nonsensical pictures (droodles). *Behav. Res. Methods* **42**, 685–691 (2010).
